# How to Value Digital Health Interventions? A Systematic Literature Review

**DOI:** 10.3390/ijerph17062119

**Published:** 2020-03-23

**Authors:** Katarzyna Kolasa, Grzegorz Kozinski

**Affiliations:** Division of Health Economics and Health Care Management, Kozminski University, 03-301 Warsaw, Poland; grzegorz.kozinski34@gmail.com

**Keywords:** digital health, mobile heath, telemedicine, value assessment, pricing and reimbursement

## Abstract

In Europe, there were almost twice as many patents granted for medical technology (13,795) compared to pharmaceuticals (7441) in 2018. It is important to ask how to integrate such an amount of innovations into routine clinical practice and how to measure the value it brings to the healthcare system. Given the novelty of digital health interventions (DHI), one can even question whether the quality-adjusted life years (QALY) approach developed for pharmaceuticals can be used or whether we need to develop a new DHI’s value assessment framework. We conducted a systematic literature review of published DHIs’ assessment guidelines. Each publication was analyzed with a 12-items checklist based on a EUnetHTA core model enriched with additional criteria such as usability, interoperability, and data security. In total, 11 value assessment guidelines were identified. The review revealed that safety, clinical effectiveness, usability, economic aspects, and interoperability were most often discussed (seven out of 11). More than half of the guidelines addressed organizational impact, data security, choice of comparator, and technical considerations (six out of 11). The unmet medical needs (three out of 11), along with the ethical (two out of 11) and legal aspects (one out of 11), were given the least attention. No author provided an analytical framework for the calculation of clinical and economic outcomes. We elicited five recommendations for the choice of DHI’s value criteria and a methodological suggestion for the pricing and reimbursement framework. Our conclusions lead to the need for a new DHI’s value assessment framework instead of a QALY approach.

## 1. Introduction

The current discussion about healthcare systems is mainly driven by the concerns for public budgets sustainability, which result from the growing life expectancy and medical needs of the aging population. In a similar fashion to many experts, the European Commission advocates for the greater adaptation of digital health solutions [1]. Many believe that digital transformation creates endless opportunities to improve health outcomes and increase the efficiency of healthcare systems [2].

The fourth industrial revolution has already become famous for the continuous introduction of multiple innovations, such as hyper-automation, human augmentation, ambient experience with virtual reality, blockchain, and 5G mobile communications. For instance, up to 26 billion units of the Internet of Things (IoT) is estimated to be installed by the end of 2020 [3]. 

However, there is still a limited number of examples of successful adaptation of digital health interventions (DHI) into the public healthcare systems thus far. In 2015, a WHO global survey on eHealth revealed that 73% of countries do not have any system in place to verify the quality, safety, and reliability of mobile health (mHealth) [4]. Consequently, the lack of legal regulations supporting the greater use of DHIs might have been one of the obstacles for digital transformation. Recent developments likely indicate some changes in that respect. In 2019, several new health policy acts were implemented. The FDA issued six different regulatory guidelines related to Software as a Medical Device (SaMD), Software in a Medical Device (SiMD), Clinical Decision Support Software, and others [5,6]. Some European authorities introduced the first guidelines in support of the introduction of DHIs into clinical pathways (the UK) or even pricing and reimbursement regulations (France, Germany) in 2019 as well [7,8,9]. Such efforts should be appreciated. We may hope that the implementation of DHIs into the public healthcare system may not only provide access for manufacturers to the additional source of financing but also ensure the safe and effective use of DHIs by patients and clinical experts and efficiency gains for the healthcare system. 

However, to achieve the above, the interests of health policymakers should further focus on the development of regulatory, clinical, and pricing and reimbursement (P&R) guidelines for digital health solutions in a similar fashion to those that are already in place for both pharmaceuticals and medical devices. It is surely debatable whether currently available regulations can be directly applicable to the DHIs’ market. 

As a contribution to the discussion regarding specifically P&R guidelines, the systematic literature review of already published assessment frameworks for digital health solutions was performed. It was to verify what kind of criteria had been used for the evaluation of DHIs thus far. It is believed that any examples can support researchers, decision-makers, and manufacturers in their efforts with the development of P&R mechanisms for DHIs. 

The cost per quality-adjusted life years (QALY) approach is considered as the gold standard for the value evaluation of pharmaceuticals. The estimation of the cost per additional health outcome defined as QALY allows payers to make pricing and reimbursement decisions by using a simple cost per QALY threshold and allocating budgets across different therapeutic areas with the same standardized approach. Namely, the QALY provides a generic approach with the added value of a given health technology being measured solely by the improvement in the mortality and quality of life. However, we may provokingly ask whether such a simplified approach that focuses solely on the clinical outcome can be adapted to the field of digital health as well. 

The systematic literature review of available assessment frameworks of digital health interventions had two-fold objectives. First, it was to describe the rationale, scope, and intended audience for specific guidelines. Second, it was to investigate the criteria and methods used in the evaluation of DHIs. The underlying assumption was that the challenge in the value assessment of DHIs was due to the complexity of the simultaneous evaluation of clinical, organizational, and economic aspects. Therefore, based on the performed literature review, we elicited a checklist with recommendations for the generation of evidence during the process of the development of digital health solutions and methodological suggestions for decision-makers for DHIs’ pricing and reimbursement framework.

## 2. Methodology 

Pubmed, Scopus, and Science Direct databases were searched for the following key phrases: “digital health interventions,” “mhealth,” “mobile health,” “telemedicine,” “health app,” and “wearables.” Each of them was paired with the term “assessment” and with one of the following: “guidelines,” “checklist,” “framework” and “consensus.” The study only included manuscripts written in English published between September 1998 and December 2019. 

In order to address the first research question, the following information was extracted: Objective, settings, intended audience, therapeutic area, and stakeholders’ engagement. The investigation with respect to the second research question was divided into two parts. 

First, the screening of included publications regarding the assessment criteria was performed. The checklist was constructed to ensure a standardized approach toward the review of each publication. The list of categories was adopted from the EUnetHTA HTA core model [10]. However, interoperability and data security were added along with usability. Overall, the list of 12-items was composed. Both authors reviewed each article independently. The examples of the assessment of each category were searched in the checklist (if available) but also in the text of each publication. In case a given category was not mentioned in a publication, the study assumed that a given criterion was not included in the proposed value assessment framework. 

Second, the study also reviewed the approach toward the weighing of attributes, if present in the article under scrutiny. The screening of included publications regarding the clinical and economic comparative analysis was conducted as well. 

## 3. Results

**Figure 1:**
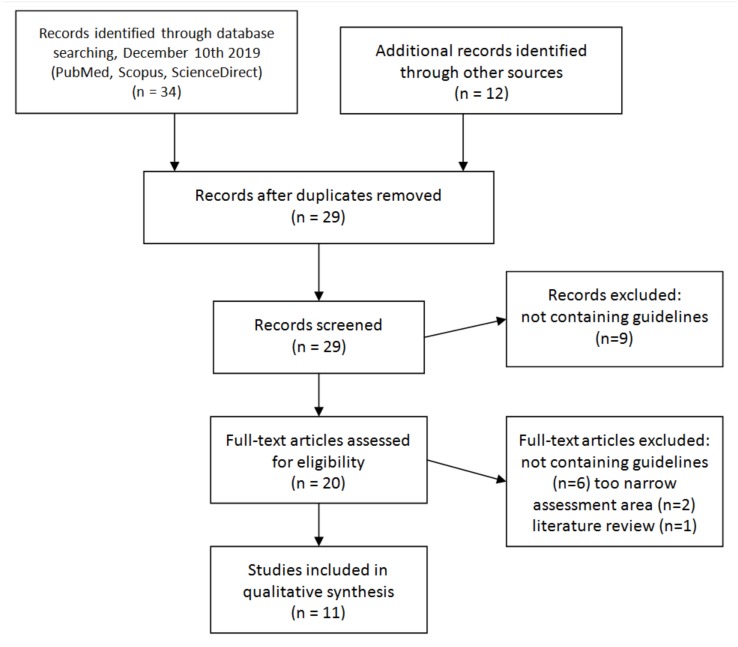
Flow chart of literature search.

The systematic literature search identified 42 records, out of which 34 were found in Pubmed, Scopus, and Science Direct databases, and 12 were added through reference search (Figure 1). A total of 17 duplications were found. Based on title and abstracts review, nine papers were excluded from further analysis as not matching inclusion criteria. In total, 20 articles were assessed for relevance. Full-text reviews revealed that a further nine publications did not fulfill the study objective. The majority of excluded studies (15) contained no guidelines or checklists. Others were limited to only one specific area of assessment (two) or reviews of available studies (one). The final review included 11 publications.

Out of the 11 discovered guidelines, three focused on telemedicine, four concerned mobile health applications, while the remaining four were generic. Among selected settings, the US, Canada, Spain, and the UK jurisdictions were chosen in seven examples [9,11,12,13,14,15,16]. Others did not consider any specific local context. The therapeutic area was selected in two cases [11,14]. It was mental, behavioral, and neurodevelopmental disorders (Table A1 and Table A2 in Appendix A). 

Three different objectives of guidelines development could be distinguished. It was to support digital solutions with value assessment (seven), support decision-makers in pricing and reimbursement, or improve reporting on the use of digital health interventions (seven; Table A1 and Table A2 in Appendix A). In five out of 11 cases, stakeholders representing multiple perspectives were included in the guidelines’ development [13,17,18,19,20] (Table A1 and Table A2 in Appendix A). The patients’ advocacy groups were mentioned in three cases while decision-makers in six [15,17,18,19,20]. 

The list of criteria varied from 10 [17] to two [15] (Table A3 and Table A4 in Appendix A). Among the most often discussed assessment domains appeared safety, clinical effectiveness, usability, economic aspects and interoperability (seven out of 11), organizational, usability, data security, and technical considerations (six out of 11). The least attention received the understanding of ethical (two out of 11) and legal aspects (six out of 11). 

The greatest number of examples appeared in the domain of organizational aspects with a key focus on the training of staff and mode of delivery. With respect to the clinical effectiveness domain, technical success rate, morbidity, and mortality were mostly mentioned. The patient’s satisfaction was proposed in four cases [15,17,18,19]. The economic domain was covered mainly with details regarding the purchasing and installment costs. Indirect costs due to the loss of productivity were mentioned once [12]. The technical aspects were mainly defined in terms of infrastructure requirements, while usability with respect to support for patients. Unmet medical needs (health problems) and technical aspects along interoperability were elaborated in a very limited manner across included guidelines.

The need for comparison against the alternative clinical practice was mentioned eight times [9,11,12,13,14,15,16,18] (Table A5 and Table A6 in Appendix A). Clinical assessments were discussed in three instances [9,15,18]. No author proposed specific guidelines for the assessment of the economic value or the weighing of criteria. 

## 4. Discussion

In our systematic literature review, we found 11 publications that fulfilled the inclusion criteria. We adopted a standardized approach to analyzing each guideline. The objective was to investigate the criteria and methods used in the evaluation of DHIs. There are two key findings important to be highlighted.

First, the results revealed the deepness of the scope of the assessment of digital health solutions. We witnessed it in the number of examples provided for the evaluation of different value domains. For instance, Kidholm et al. offered at least seven examples of how to assess clinical aspects (18). A similar number of items was presented by Hailey et al. with respect to the economic consequences of DHI implementation (11). There were 13 different types of patient and social aspects in the assessment framework for digital health solutions mentioned in the NICE guidelines (15).

Second, despite such great attention given to some specific aspects, there were other value domains left without any attention. An example can be the lack of consideration given towards the unmet medical needs (health problems; three). The stakeholders’ involvement was reported only in four cases. None of the reviewed guidelines proposed a methodological approach to the calculation of the economic value. 

Both conclusions should be considered with caution due to at least three reasons. First, we managed to identify only the guidelines with a specific focus on telemedicine and mobile health applications. Hence, our study may not be generalizable to other forms of digital health solutions, such as digital clinical decision support applications. Second, despite the thorough review of included publications, there remains the risk that we missed some important aspects mentioned by their authors in each of 12 value domains. Finally, our approach was limited to pre-defined value attributes. Thus, there might be other vital signs of the role of digital health solutions to be assessed but not covered by our study.

Despite the above limitations, the findings of our systematic literature review allow us to construct a short checklist with five recommendations to be considered for the generation of evidence during the process of development of digital health solutions by manufacturers and methodological suggestion for a future framework for the DHIs value assessment in the pricing and reimbursement processes. Table A7 and Table A8 provide further details on how each of the identified guidelines performed against our checklist. Below, we present each recommendation with examples from the systematic literature review as well as other studies.

### 4.1. Choice of Comparator

The alternative treatment option is understood as the method of care to be replaced with a digital health solution. It determines the unmet medical needs that DHI should address by its value to be delivered to the healthcare system. Limited attention was given to the choice of comparator across the reviewed guidelines. Out of the 11 guidelines found, there were only two that addressed that issue [11,15] (Table A7 and Table A8 in Appendix A). 

It is a surprising finding if one takes into consideration the fact that the value of new health technology is determined by the forgone outcome to be delivered by the alternative course of action. Hailey et al. chose to address that challenge with a specific definition of the comparator: “there will generally be a need for a description and measurement of the technology that will be in place if the telemedicine application is not adopted.” Given the organizational character of changes in the clinical pathway introduced by DHI, the alternative course of action might not be related to clinical improvements but organizational aspects. Therefore, it is even more important to address the challenge of the comparator’s identification with due consideration. An example here can be Jane E. Sisk’s elaboration on the full range of potential alternatives for the service of a radiologist supported by telemedicine: “(1) the patient could travel to the radiologist’s location for the imaging study and consult, and then return home; (2) the imaging could be performed at the patient’s home health facility, and the radiologist could travel round trip to consult; (3) express mail could transport the image to the radiologist and the reading back to the home health facility; or (4) telemedicine could substitute for express mail.” Similar alternatives apply to the process of obtaining counseling for a patient at a distance from the therapist: “(1) the patient could travel round trip; (2) the therapist could travel round trip; or (3) telemedicine could televise audiovisual information between the two people at their original locations.” 

In conclusion, it is extremely vital to investigate what is the current standard of care by addressing the question: What if the digital solution was not available? The unmet medical needs and/or organizational inefficiency should be addressed in the process of identifying the appropriate comparator.

**Recommendation 1:** The value of a digital health solution should be determined by the incremental advantage it can deliver compared to the current standard of care

### 4.2. Multi-stakeholders Perspective

The multi-stakeholders’ perspective is understood as the simultaneous assessment of DIHs’ benefits from the view of patients, clinicians, payers, and healthcare managers. Among the included studies, there were just two publications [15,20] that covered all four stakeholders’ perspectives and six that limited the analysis to only three or two [9,11,12,13,18,19]. The remaining ones considered just one viewpoint [14,16,17] (Table A7 and Table A8 in Appendix A).

Interestingly, the checklists developed on multi-stakeholders’ involvement provided a larger number of criteria compared to those prepared by authors alone. Among frequently consulted experts were decision-makers, manufacturers, and academic researchers. The most advanced approach to the selection of assessment criteria was the one conducted by Agarwal et al. within the mEra project [19]. The process involved a systematic literature review, interviews with experts, and stakeholders’ consultation during a three-day meeting. Interestingly enough, the mEra checklist encompasses the longest list of criteria, with 16 items on their checklist [19]. In a similar fashion, there were two rounds of consultations organized in the EU project, which included both workshops and online questionnaire studies for stakeholders [20]. The checklist included as many as 13 items. Although the greater number of criteria does not guarantee better assessment, it surely allows for more objective and robust estimation of the value of a given DHI. It is difficult to anticipate a successful implementation of specific digital health solution if it does not meet the expectations of all its end-users. 

Given that there are multiple DHI beneficiaries, it is crucial to ensure a robust assessment of the potential impact from multiple perspectives. Given the multi-beneficiaries dimension of DHIs, the value assessment should quantify the incremental differences it brings to all its recipients. Since QALY focuses on the patient’s perspective alone, the adaptation of such an approach may lead to an underestimation of the value of DHIs. In the process of DHIs development, manufacturers should investigate the unmet needs of clinicians, patients, payers, and managers responsible for the organization of healthcare.

**Recommendation 2**: The value of a digital health solution should quantify incremental differences it delivers to all beneficiaries, including patients, clinicians, payers, and healthcare managers.

### 4.3. Organizational Impact 

The organizational impact is understood as the healthcare system preparedness to consume efficiency gains from DHIs’ implementation. Among the included studies, there are four publications that did elaborate on that aspect of value generation [11,15,18,19]. However, this aspect was limited to the discussion about preconditions for DIHs’ implementation. None of the reviewed guidelines proposed a feasibility study or a quantification of the potential efficiency gains and losses (Table A7 and Table A8 in Appendix A).

The complexity of organizational consequences assessment of DHIs’ implementation depends on both technical characteristics of a specific technology and the unmet medical needs that should be addressed. In fact, the value of digital health solutions must be measured by its technical adaptability to clinical pathways. In many instances, the success of DHIs’ implementation depends entirely on the healthcare system’s capacity to adopt innovation. Therefore, it is key to accurately quantify the needs of both investment and disinvestment. Healthcare professionals’ and patients’ preparedness for a change of clinical pathways are further crucial success factors. Unless a specific training and understanding of DHIs’ benefits are ensured, preparedness may constitute a substantial barrier to the successful launch of a new digital health solution. On that note, we must remain mindful of the learning curve of end-users’ experience. A very comprehensive set of examples of the organizational impact is provided by Hailey et al., who mention staff training, delivery arrangements, and travel time for patients and healthcare professionals [11]. 

The implementation of digital health solutions into the clinical practice will certainly influence the perspective on the integrated healthcare model. In order to consume all the benefits of DHIs, we must approach the healthcare system from a holistic perspective. A new integrated healthcare system needs to account for DHIs’ efficiency gains in both clinical and organizational dimensions. Efficiency gains can only be achieved if data generated by DHIs will be incorporated by healthcare professionals in their decision-making processes. If that happens, we will observe many examples of remote video or chat care, which will free up clinicians’ calendars to allow them to take care of more severe cases that require their personal attention and presence.

In summary, the value of a DHI depends not only on its technical capacity but also on its successful implementation, which might be difficult to embrace with the cost per QALY approach. Therefore, there is a need for a study of the organizational impact with respect to both infrastructure investment and disinvestment, but also the changes in clinical pathways resulting from DHIs’ implementation.

**Recommendation 3:** The value of digital health solutions should be conditional on the healthcare system preparedness to consume efficiency gains and assurance that data generated by DHI will be accessible by healthcare professionals.

### 4.4. Multidimensional Outcome

The multidimensional outcome is understood as the quantification of the clinical, organizational, behavioral, and technical performance of a digital health solution. Among identified guidelines, there was not a single publication that covered all four categories. At the same time, there were eight articles that limited their attention to not more than three aspects [9,11,13,14,15,16,17,18,19,20] (Table A7 and Table A8 in Appendix A). 

Unfortunately, the available checklists neglect to describe how different dimensions of health outcome interconnect with each other and define the value of digital health solutions. For the sake of simplicity, let us focus on the example of a hypothetical mobile application designed to improve adherence and ensure dose optimization of medications. Assuming that it does provide remote monitoring for healthcare professionals, the value this application delivers may stretch beyond the improvement of the treatment outcome (clinical dimension). Moreover, it may decrease healthcare resource consumption by the elimination of follow-up visits (organizational dimension). Surely, the patient’s understanding and confidence in the usefulness of DIH plays a role as well (behavioral dimension). Furthermore, in order to ensure treatment safety, the technical reliability of DIH must be validated (technical dimension). Importantly, the latter was already introduced in Kidholm et al. [18], but no author discussed such multidimensional nature of outcome for digital health solution (18). Therefore, we should underline that the value of DHIs’ has multiple dimensions beyond clinical outcomes. As such, the QALY approach might underestimate the real impact of the implementation of DHI into clinical practice. After we have identified all beneficiaries of DHIs, it will be easier to list all the potential dimensions of DHIs’ value, which may include both clinical and organizational outcomes. 

**Recommendation 4**: The value delivered by digital health solutions should consider multiple dimensions such as clinical, organizational, behavioral, and technical.

### 4.5. Interoperability 

Interoperability is understood as the ability of digital health solutions to be connected to other data sources. The identified guidelines touched upon the aspect of interoperability in a very brief fashion. Out of the 11 publications, there were eight that addressed this aspect [9,13,14,16,17,18,19,20] (Table A7 and Table A8 in Appendix A). 

Following the concept of an integrated healthcare system, it is vital to ensure connectivity across health data sources. Therefore, the value of DIH should not be assessed solely on its technical sophistication and unmet medical needs but also on the data it generates, along with methods that allow the data to be incorporated in the decision-making processes. As such, the concept of interoperability should be the key component of the DIHs’ development. Interoperability does not only depend on technical specifications of DIHs but also on a healthcare system’s capacity to receive and analyze new data streams. Despite the number of global initiatives [21,22], there might be structural differences across healthcare systems organization in different jurisdictions. Therefore, any feasibility study of data connectivity must be conducted on the country level in order to objectively and rightly assess the value of specific digital health solutions. Interoperability is a complex challenge, as it can be defined in multiple dimensions such as structural interconnectivity (data format standards), semantic interconnectivity (ability to exchange and interpret the data), and organizational interconnectivity (legal and organizational facilitators of data exchange). Therefore, interoperability is the key concept for integrated healthcare models, which should be taken into consideration during the phase of digital health product development and as part of its value assessment. Most importantly, interoperability’s scope must be defined separately by each jurisdiction to ensure its relevance for country-specific settings.

**Recommendation 5:** Connectivity to other data sources must be considered in the evaluation of a digital health solution 

### 4.6. Value Aggregation Function 

The choice of the value function is crucial to define a unique value score for each DIH. The systematic literature review leaves unanswered the challenge of value criteria weighing and their aggregation. 

It is a surprising finding, especially if one considers the growing interests in the adaptation of the multi-criteria decision-making (MCDA) approach in pricing and reimbursement processes for pharmaceuticals across different jurisdictions. The MCDA methodology does allow for incorporating multiple, even conflicting objectives, into a single decision-making choice. It happens by assigning different weights to criteria according to the preferences of the chosen stakeholder group and the calculation of aggregated value scores for each health technology. There are multiple publications that provide the appropriate methodology in that respect. However, it is not our intention to review it. 

As a consequence of the lack of approach to criteria weighing and aggregation, it is challenging to compare the aggregated value score of different DHIs against each other. Since the P&R decision for a given health technology never happens in isolation from other choices, there is a need for an objective and robust framework that would not only allow us to assess the value of a single DHI but would also allow us to compare this value against other. Many scholars believe that the MCDA approach can facilitate this process.

**Methodological suggestion**: Given the multiple criteria to be considered in the pricing and reimbursement process, each value attribute should be weighted in any calculations based on the preferences of chosen stakeholder groups, and the aggregate value score for each DHI should be estimated. 

## 5. Conclusions

The era of digitalization in healthcare brings with it a cultural shift. We should understand that digital health solutions will not decrease the need for clinical expertise. On the contrary, it will support the work of healthcare professionals and make clinical decision-making easier and more effective. Therefore, it is valid to discuss whether digital health solutions should be funded by the public budget as is the case with other health technologies. The grounds for this discussion is that digital health solutions can bring at least two types of benefits: (1) clinical gains achieved by more effective, and (2) safe treatment and efficiency gains achieved by the better organization of the healthcare system. 

Despite the growing understanding of the opportunities offered by DHIs, there still is a lack of methodological advancements to support decision-makers with the funding mechanisms. Our systematic literature review indicates that there is room for improvement regarding the scope of criteria to be taken into consideration in the value development of DHs and in the methodological approach adopted to measure the incremental benefits of DHIs during the P&R processes. 

We proposed five recommendations for the generation of evidence to be considered in the process of digital health solutions’ development and suggestion for the methodological approach to be adopted in DHIs’ pricing and reimbursement. The latter has special importance as our systematic literature review revealed that there is not a single value assessment framework that will allow for the evaluation of the multidimensional outcome delivered for multi-stakeholders. 

In many jurisdictions, there already exist P&R regulations focused on the value assessment for pharmaceuticals, usually based on health technology assessment (HTA). The interest to adopt HTA for the assessment of non-pharmaceutical technologies is growing across various healthcare systems. HTA allows for a simultaneous assessment of the clinical and economic benefits of a health technology. It mainly proposes the cost per QALY approach. However, there are distinct differences between pharmaceuticals and digital health solutions that must be taken into consideration in the development of a new approach for the value assessment framework of the latter. Some distinct features of digital health may even make HTA’s framework redundant. The value of digital health solutions does not only depend on clinical and economic aspects but also on technical features, perceived benefits for healthcare managers, willingness to use by end-users, and finally, the healthcare system’s capacity to benefit from the innovation. Moreover, the determination of the appropriate alternative course of action may be different for DHIs than pharmaceuticals. 

Nevertheless, we should ensure that digital health interventions meet similar requirements for the generation of evidence as do other health technologies. Given the multidimensional character of DHIs’ benefits, our five recommendations indicate that the QALY approach might be both challenging and irrational. There is a need for further considerations toward the development of the value assessment framework for DHIs. Hopefully, our suggestions can be regarded as the first step in this direction. Certainly, our results must be treated with caution and require further discussion among diverse stakeholders. The final list of DHIs’ value criteria should be developed in the form of a consensus across all interest groups. At the same time, further research is necessary to specifically develop an appropriate aggregated value function so as to ensure the robustness and scientific rigor for the assessment and comparison of digital health solutions, as it happened for other health technologies such as pharmaceuticals.

## Appendix A

**Table A1:** General information.

	An Assessment Framework for Telemedicine Applications [11]	mHealth Assessment: Conceptualization of a Global Framework [17]	A Proposed Framework for Economic Evaluation of Telemedicine [12]	A Model for Assessment of Telemedicine Application: MAST [18]	Guidelines for Reporting of Health Interventions Using Mobile Phones: Mobile Health (mHealth) Evidence Reporting and Assessment (mERA) Checklist [19]	How we Assess Health Apps and Digital Tools—NHS Digital Guidelines [13]
Objective	Support with value assessment of digital solutions	Support with value assessment of digital solutions	Support with value assessment of digital solutions	Support of decision-makers	Improve the reporting of mobile health interventions	Support of decision-makers
Settings	Canada	NA	US	NA	NA	England
Authors of guidelines	Hailey D.; Jacobs P. et al.	Bradway M.; Carrion C. et al.	Sisk J. E.; Sanders J.H.	Kidholm K.; Ekeland A. G. et al.	Agarwal S.; LeFevre A. E. et al.	NHS Digital
Intended audience	Decision-makers	Decision-makers, manufacturers, general public	Decision-makers	Decision-makers	Decision-makers, general public	Manufacturers
Therapuetic area	F01-F99 Mental, Behavioral and Neurodevelopmental disorders	NA	NA	NA	NA	NA
Stakeholders involved	NA	Decision-makers, manufacturers, general public	NA	Service providers, users, and decision-makers	Academic researchers, manufacturers, decision-makers, representatives of several WHO departments	Industry experts with appropriate qualifications in areas of data protection, accessibility, and technical stability
Methods of criteria selection	NA	Expert consultations	NA	Expert consultations, user consultations, systematic literature review	Expert consultations	Expert consultations

**Table A2:** General information—continued.

	App Evaluation Model—American Psychiatric Association [14]	Evidence Standards Framework for Digital Health Technologies [15]	Andalusian mHealth Strategy—Use Case [16]	Medical Device Evaluation by the CNEDiMTS (Medical Device and Health Technology Evaluation Committee) [9]	Report of the Working Group on mHealth Assessment Guidelines [20]
Objective	Support with value assessment of digital solutions	Support of decision-makers	Support with value assessment of digital solutions	Support with value assessment of digital solutions	Support with value assessment of digital solutions
Settings	US	England	Spain	France	
Authors of guidelines	American Psychiatric Association	National Institute for Health and Care Excellence	Agencia de Calidad Sanitaria de Andalucía	French National Authority for Health (HAS)	NA
Intended Audience	American Psychiatric Association members	Decision-makers, manufacturers	Manufacturers, general public	NA	Decision-makers, general public
Therapeutic area	F01-F99 Mental, Behavioral and Neurodevelopmental disorders	NA	NA	NA	NA
Stakeholders involved	NA	Decision-makers	NA	NA	Patients, industry experts, decision-makers, academic researchers, general public
Methods of criteria selection	NA	Expert consultations	NA	Systematic literature review	Stakeholders group activities, online questionnaires

**Table A3:** Criteria assessment.

	An Assessment Framework for Telemedicine Applications [11]	mHealth Assessment: Conceptualization of a Global Framework [17]	A Proposed Framework for Economic Evaluation of Telemedicine [12]	A Model for Assessment of Telemedicine Application: MAST [18]	Guidelines for Reporting of Health Interventions Using Mobile Phones: Mobile Health (mHealth) Evidence Reporting and Assessment (mERA) Checklist [19]	How We Assess Health Apps and Digital Tools—NHS Digital Guidelines [13]
Health problem and current use of technology	Current standard of care	Designation of mHealth solutions by intended use	NA	Health problem	NA	NA
Safety	Risk of wrong diagnostic Risk of wrong management Delayed treatment	Security Electricity related	NA	Clinical safety (patients and staff) Technical safety (technical reliability)	NA	NA
Clinical effectiveness	Performance under carefully applied and monitored protocols Length of hospital stay and numbers of prescriptions Health-related effects	Behavioral change (lifestyles, self-management) Decrease prevalence, increase awareness Disease prevalence and incidence QALYs gained	Increase in volume of health outcomes Improvement in learning curve of medical professionals	Effects on mortality Effects on morbidity Effects on health-related quality of life (HRQL) Behavioral outcomes Usage of health services Understanding of information Confidence in the treatmentself-efficacy	NA	What does it claim to do vs. what does it actually do?
Patient and social aspects	NA	Empowerment and satisfaction Improved access to care (waiting lists) Increased productivity (decreased disability rates, lost workdays)	Patient time	Satisfaction and acceptance Ability to use the application Access and accessibility Empowerment	Patient satisfaction	NA
Economic	Costs of the required infrastructure? Are all needs fulfilled by the use of purchased equipment? Other costs involved Cheaper equipment options Purchase Maintenance Operational costs	Costs (devices, infrastructure, network)	Cost of treating a patient Changes in the productivity of health professionals Changes in transportation costs and supplies Capital costs Travel costs	NA	Cost assessment	Cost assessment
Legal	NA	NA	NA	National and regional legislation accordance	NA	NA
Ethical	NA	NA	NA	Ethical issues	NA	NA
Organizational	Staff training Delivery arrangements Travel time for patients and health-care professionals Consultation with staff	Training and coordinated care	The possibility of expanded indications	Process Structure Culture	Intervention delivery Intervention content Limitations for delivery at scale Contextual adaptability User feedback Access by individual participants	NA
Usability	Number of sites Possible back-up options Operator and coordinator Staff aspects Is wider consultation required?	User guides User support	NA	NA	Access to all its participants Barriers to adoption	User can understand how the app works User experience standards Web Content Accessibility Guidelines 2.1 Updates after user feedback
Data security	NA	Security standards	NA	Data loss Safety	Hardware and software safety Data reception Data storage and access Data sharing protocols	How data is stored? Open Web Application Security Project Standards Tests with OWASP Mobile Security Testing Guide (MSTG)
Interoperability	NA	Integration with existing medical system	NA	Integration with different systems	Connection with Health Information Systems (national or local) Data transfer standards	NHS England’s Open API policy Global interoperability standards and NHS Interoperability Toolkit
Technical aspects and stability	Communication carrier Required equipment Number of telephone lines	Infrastructure	NA	NA	Adoption inputs Replicability Platform description Software and hardware aspects	Reporting technical issues Patient data after users stop using it Updates issues NICE Evidence

**Table A4:** Criteria assessment—continued.

	App Evaluation Model—American Psychiatric Association [14]	Evidence Standards Framework for Digital Health Technologies [15]	Andalusian mHealth Strategy—Use Case [16]	Medical Device Evaluation by the CNEDiMTS (Medical Device and Health Technology Evaluation Committee) [9]	Report of the Working Group on mHealth Assessment Guidelines [20]
Health problem and current use of technology	NA	NA	NA	NA	NA
Safety	NA	Potential harm Consequences in case of fail to preform Used by vulnerable groups Used with/without regular support	Possible risks for patient safety Known risks and adverse events (near misses)	Safety	Safety
Clinical effectiveness	NA	NA	Scientific evidence and the type of sources (systematic revisions, clinical practice guidelines, peer-reviewed articles, consensual protocols, experts’ consensus, etc.)	Morbidity Mortality Quality of life criteria Acceptability and patient satisfaction Access to treatment Treatment quality Professional practices	NA
Patient and social aspects	NA	Feelings/experiences and comfort Professional-patient interaction Timeliness and convenience Overall satisfaction Preference between face-to-face and telemedicine Professional Competence/ Personal Manner Technological informativeness Usefulness Confidence (in a treatment) Ability to use the application Empowerment Access Self-efficacy	NA	NA	NA
Economic	NA	NA	The estimated consumption of resources with economic cost	Costs	NA
Legal	NA	NA	NA	NA	NA
Ethical	NA	NA	Identification of possible ethical conflicts Ethical considerations specification	NA	NA
Organizational	NA	NA	NA	Reliability	NA
Usability	App’s business model Advertisement Customizable features? Users with impaired vision Ease of use	NA	NA	NA	Accessibility Usability/desirability
Data security	Data collection Privacy policy Data removal Data sharing Local or cloud storage Data encryption HIPAA recommendations	NA	NA	NA	Privacy
Interoperability	Connection with Electronic Health Records (EHR) Sharing data Exporting user data	NA	NA	Interaction with other devices Data collection transmission and data processing	Interoperability
Technical aspects and stability	NA	NA	NA	CE marking	Technical stability

**Table A5:** Comparative assessment.

	An Assessment Framework for Telemedicine Applications [11]	mHealth Assessment: Conceptualization of a Global Framework [17]	A Proposed Framework for Economic Evaluation of Telemedicine [12]	A Model for Assessment of Telemedicine Application: MAST [18]	Guidelines for Reporting of Health Interventions Using Mobile Phones: Mobile Health (mHealth) Evidence Reporting and Assessment (mERA) Checklist [19]	How we Assess Health apps and Digital Tools—NHS Digital Guidelines [13]
Comparator	Compare performance with a pilot project	NA	Consideration of expected effects in means of health benefits	Comparing application with relevant alternatives in	NA	NA
Comparative clinical analysis	Compare performance with a pilot project	NA	Consideration of expected effects in means of health benefits	Effects on mortality Effects on morbidity Effects on health-related quality of life (HRQL) Behavioral outcomes Usage of health services	NA	Proving that app improves patient’s health and meets their needs independent research evidence
Comparative economic analysis	NA	NA	Must consider all possible alternatives Costs should consider changes in all aspects compared with alternatives Predefined decision rules A consideration of payment options	Comparison from a cost and health consequences perspectives The amount of resources and their prices Changes in use and effectiveness	Conducted in comparison with at least two alternatives Should be reported with the 24-item CHEERS statement Should be precise and include aspects such as currency, conversion, and price date	NA

**Table A6:** Comparative assessment—continued.

	App Evaluation Model—American Psychiatric Association [14]	Evidence Standards Framework for Digital Health Technologies [15]	Andalusian mHealth Strategy—Use case [16]	Medical Device Evaluation by the CNEDiMTS (Medical Device and Health Technology Evaluation Committee) [9]	Report of the Working Group on mHealth Assessment Guidelines [20]
Comparator	Peer-reviewed evidence Users’ feedback to support it	Comparator should be a care option that is reflective of the current care pathway	NA	Standard of care	NA
Comparative clinical analysis	Peer-reviewed evidence users’ Feedback to support it	Observational or quasi-experimental studies with behavioral outcomes or RCT	Tests done to representative users Outcomes are analyzed Problems detected during tests should be corrected before the launch of the App	The preclinical phase (Technology development Clinical feasibility phase (Feasibility studies, Studies demonstrating clinical benefit) MD development (Post-market clinical follow-up (PMCF), Post-registration inclusion studies (PRS))	NA
Comparative economic analysis	NA	Budget impact analysis Cost-consequence analysis or cost-utility analysis	NA	NA	NA

**Table A7:** Short checklist.

	An Assessment Framework for Telemedicine Applications [11]	mHelath Assessment: Conceptualization of a Global Framework [17]	A Proposed Framework for Economic Evaluation of Telemedicine [12]	A Model for Assessment of Telemedicine Application: MAST [18]	Guidelines for Reporting of Health Interventions Using Mobile Phones: Mobile Health (mHealth) Evidence Reporting and Assessment (mERA) Checklist [19]	How we Assess Health Apps and Digital tools—NHS Digital Guidelines [13]
Recommendation 1: The value of digital health solution is determined by the incremental advantage it can deliver compared to the current standard of care.						
**What is the current standard of care to be replaced with digital health solution?**	X	-	-	-	-	-
Recommendation 2: The value of digital health solution depends on perceived benefits by patients, clinicians, payers, and healthcare managers.						
Has the benefits of each stakeholder group been taken into consideration?						
**Patients**	X	X	X	X	X	X
**Clinicians**	-	-	-	-	-	-
**Payers**	X	-	X	X	X	X
**Healthcare managers**	-	-	-	X	-	-
Recommendation 3: The value of digital health solution is conditional on the healthcare system preparedness to consume efficiency gains.						
**What are preconditions for DIH implementation?**	X	-	-	X	X	-
**Has there been any feasibility study to assess potential efficiency gains and losses?**	-	-	-	-	-	-
**Has there been any quantification of potential efficiency gains and losses?**	-	-	-	-	-	-
Recommendation 4: The health outcome delivered by digital health solution has multiple dimensions such as clinical, organizational, behavioral, and technical.						
Has each dimension of the health outcome been considered?						
**Clinical**	X	X	-	X	-	X
**Organizational**	X	X	-	X	X	-
**Behavioral**	X	-	-	-	-	X
**Technical**	-	-	-	-	X	X
Recommendation 5: In the development of digital health solution its connectivity to other data sources must be ensured.						
**Has interoperability been taken into consideration?**	-	X	-	X	X	X

**Table A8:** Short checklist—continued.

	App Evaluation Model—American Psychiatric Association [14]	Evidence Standards Framework for Digital Health Technologies [15]	Andalusian mHealth Strategy—Use Case [16]	Medical Device Evaluation by the CNEDiMTS (Medical Device and Health Technology Evaluation Committee) [9]	Report of the Working Group on mHealth Assessment Guidelines [20]
Recommendation 1: The value of digital health solution is determined by the incremental advantage it can deliver compared to the current standard of care.					
**What is the current standard of care to be replaced with digital health solution?**	-	X	-	-	-
Recommendation nr 2: The value of digital health solution depends on perceived benefits by patients, clinicians, payers and healthcare managers.					
Has the benefits of each stakeholder groups been taken into consideration?					
**Patients**	X	X	X	X	X
**Clinicians**	-	X	-	X	X
**Payers**	-	X	-	-	X
**Healthcare managers**	-	X	-	-	X
Recommendation nr 3: The value of digital health solution is conditional on the healthcare system preparedness to consume efficiency gains					
**What are preconditions for the DIH implementation?**	-	X	-	-	-
**Has there been any feasibility study to assess potential efficiency gains and losses?**	-	-	-	-	-
**Has there been any quantification of potential efficiency gains and losses?**	-	-	-	-	-
Recommendation nr 4: The health outcome delivered by digital health solution has multiple dimensions such as clinical, organizational, behavioral, and technical.					
Has each dimension of the health outcome been considered?					
**Clinical**	X	X	-	X	X
**Organizational**	-	X	-	X	-
**Behavioral**	-	X	-	-	-
**Technical**	-	-	X	-	X
Recommendation nr 5: In the development of a digital health solution its connectivity to other data sources must be ensured.					
**Has interoperability been taken into consideration?**	X	-	X	X	X
